# Medical Opinion and Movement

**Published:** 1910-09-17

**Authors:** 


### Medical Opinion and Movement.

### Orthostatic Albuminuria.

Some two or three years ago Dr. Jehle developed a mechanical theory to explain the phenomenon of orthostatic albuminuria and supported his opinions by cases in which the condition was associated with lordosis of the spine, the idea being that the lordosis caused a mechanical pressure and consequent stasis in the inferior vena cava. Dr. Vorphal now publishes a report of a case in which this condition of orthostatic albuminuria was associated with a scoliosis of the vertebral column. The patient was a young girl aged twelve, complaining of headache, pains in the back, and general fatigue and anorexia. There was albuminuria which completely disappeared on resting, but reappeared within twelve minutes of assuming the erect position. There were marked scoliosis and slight lordosis of the vertebral column, and these were most accentuated in the erect position. In the sitting position, in which the lordosis disappeared but the scoliosis was little affected, there was still albuminuria. Careful examination of the urine, etc., failed to elicit any other morbid sign. Catheterisation of the ureters revealed the fact that the albumin only came from the right kidney. The author was led to conclude, therefore, that the albuminuria was due to a dragging exerted on the renal vessels from the scoliosis in the erect position.

### Oxygen in Abdominal Operations.

The use of oxygen has been advocated by Dr. Thiriar in all manner of surgical procedures by reason of the favourable action it exerts on the activity of the leucocytes and its power to accelerate diapedesis. On the same grounds Drs. Weiss and Sencert have tried its effects in cases of general peritonitis. They have followed the treatment now for the last three years, and are much impressed with the results. A report of their work appears in the *Revue de Chirurgie*. In a hundred cases of appendicitis operated upon during an acute attack they have not had a single failure, and they claim to have been able to save eleven patients out of sixteen in a very serious condition by their method of carrying a continuous current of oxygen into the abdominal cavity. And even in cases that have proved fatal owing to further complications by persistence of the traumatic shock, the authors have observed, as a result of the treatment, a marked improvement in the pulse and general condition of the patient. The method of procedure is as follows: The abdomen being opened, and whatever necessary in the way of surgical intervention being carried out, it is washed out with hydrogen peroxide, rough handling being carefully avoided. The abdominal wall is then sutured and drainage tubes inserted, these varying somewhat according to indications. The authors advise, for general cases, a hypogastric, an iliac, on one or both sides, and possibly also a lumbar tube. A current of oxygen is then passed through the hypogastric tube from an ordinary oxygen cylinder. The gas emerges by one of the lateral tubes, the lower end of which is immersed in an antiseptic solution. Purulent serum and tissue débris are carried out through the tube with the gas. The current of oxygen should be continued for some days till the patient is quite out of danger, and the drainage tubes should remain in as long as there is any exudate.

### The Pathology of Suicide.

According to an interesting contribution to the *Wiener Klinische Wochenschrift* by Dr. J. Bartel, there is a close association between suicide and the *status lymphaticus*. His attention was drawn to the matter by observing this condition in three autopsies he carried out on suicides. The subjects were a young man aged eighteen and two young girls aged nineteen and twenty-one. All had enjoyed apparently good health, and in each case there were well-marked signs of lymphatism, including considerable hypertrophy of the thymus gland. A further study of the subject is based upon 122 autopsies of suicides, of which sixty-seven were male and fifty-five female. The most constant anomalies were relative to an exaggerated development of the thymus and the lymphatic systems, and this was especially marked in the young subjects, so that in the majority of cases in this category there was present an actual *status lymphaticus*. Other points noted were a deficiency in the development of the arterial system in proportion to the size of the individual, and a frequent colloid degeneration of the thyroid gland. The ovaries were almost always large, and usually showed the presence of numerous follicular cysts. Anomalies in development were also common.

### Conjunctival Secretions.

Little attention has been hitherto devoted to the practical utility from a clinical point of view of a cytological and microchemical examination of the conjunctival secretions. Dr. Ch. Lafon has attempted to supply this deficiency, and publishes an account of his work in the *Annale d'Oculist*. He finds that generally in cases of conjunctivitis, and especially in catarrhal or purulent forms, the polynuclear leucocytes are the predominant factor, amounting to 94 to 98 per cent. of the total cellular elements. He notes, however, certain exceptions: subacute conjunctivitis with the diplobacillus of Morax is characterised by a certain degree of lymphocytosis, amounting to 20 per cent., against 77 per cent. of polynuclears. This lymphocytosis is still more marked in follicular conjunctivitis, in which it attains to the figure 56 per cent., while in trachoma, which is otherwise easily confused with the latter, it only amounts to 12 per cent. The eosinophilia of "spring conjunctivitis," amounting to 54 per cent., is even more typical, and has enabled the author to make an exact diagnosis in two doubtful cases. On the other hand, certain conditions of conjunctival irritation without infection, due to mechanical or chemical action, may give the appearance of an infective conjunctivitis, but in such cases the conjunctival secretion shows the presence of desquamated epithelial cells and not leucocytes. Thus, in the case of a student who, having received some tuberculous pus in the eye, used antiseptic lotions too freely, it was easy to demonstrate that the consequent conjunctivitis was due to the intense reaction set up by the antiseptic, and not of a tubercular nature. The author also employs these cytological examinations to determine whether the conjunctival sac is infected or not previous to some operation on the eyeball, such as the operation for cataract. From his experience of thirty cases of cataract extraction he concludes that for such an operation the conjunctival secretion should not show polynuclear leucocytes in more than one field in five.

### A New Operation for Prolapse.

An ingenious but complicated operation for the relief of uterine prolapse has been devised by Dr. M. L. Harris, and described in the *Journal of the American Medical Association.* The procedure involves meddling with a good many important structures, but if it proves effective, the risks incidental to it may be worth running. He opens the abdomen in the Trendelenburg position, packs off the intestines, and incises the peritoneum along the tendon of the psoas parvus, which forms a white band along the psoas magnus. The external iliac vessels are pushed off the inner border of the latter muscle, and the genito-crural nerve is also carefully avoided; then the tendon of the psoas parvus is divided at its insertion into the pectineal eminence. The finger is pushed outwards under the infundibulo-pelvic ligament until it appears under the peritoneum in a small space anterior to the internal iliac vessels external to the ureter. Here the peritoneum is again divided, a curved forceps inserted and pushed through to catch the end of the divided tendon, which is then pulled out. The two psoas parvus tendons thus treated are then sutured to the posterior surface of the cervix. The result is to hold up the uterus in the pelvis, with the cervix well back, by an active muscular support. If the parvus be absent, a small portion of the magnus may be used.

### Ectopic Gestation in Australia.

The *Australasian Medical Gazette* contains two papers on this subject by authors whose experience of the condition is amazingly extensive. Mr. Gordon Craig, who practised on the outskirts of Sydney for fourteen years before he became a consultant, met with as many as 109 cases during that time, a number which seems astounding for a general practice, however extensive. His explanation of this frequency is that pelvic inflammation, the result of miscarriages or venereal disease, is very common in the industrial centre where he lived. The chief point of interest in his communication is the stress which he lays upon a sign which he has never yet failed to elicit, except in some of the patients who had a simple hæmatocele only. This is a tenderness of the cervix, caused especially by putting one finger on the external os and slowly lifting the uterus upward and to either side, so as to produce tension on the broad ligaments. In one patient he noticed a spasm of pain crossing the face as he did this, though the woman was so nearly dead of hæmorrhage as to be comatose and quite unconscious; a timely operation saved her. Dr. Taylor Young writes his paper from the standpoint of the operator, basing it upon a series of 108 cases. The keynote of his paper, naturally enough, in view of his large experience, is the advice never to forget ectopic gestation, unless it can be practically excluded, rather than to remember it only when the diagnosis stares one in the face. General uneasiness, constant slight pain, and a feeling that things are not right are often to be elicited by careful questioning, he says, and are valuable aids to diagnosis. It is worth noting that he discountenances the Trendelenburg position for the operation.

### Mosquito Nets and Health.

An important point was raised at the recent British Medical Association meeting by Dr. G. D. Whyte, who has been analysing the air inside and outside the mosquito curtain as used for protection of sleepers in malarial districts. He finds that the percentage of carbon dioxide is practically the same under the net as in the atmosphere of the room outside it. This holds good whether doors and windows are open or shut. In this respect the net is absolved of any evil influence. It is otherwise when the organic content of the air is analysed, for this is twice as great inside the curtains as outside them. He regards the use of the net as detrimental to health for this reason; and for the tuberculous the disadvantage is so great that he prefers to rely on citronella or other insecticide for warding off the malaria-carriers. He recognises fully the enormous gain of using nets for preventing insect-borne diseases, but points out that there is a corresponding disadvantage in the accumulation of organic matter which is thus encouraged. It is probably this organic matter which renders the mosquito-curtain so excessively irksome on hot and airless nights in the tropics, when the temptation is so great to throw it aside and risk a few bites for the sake of getting off to sleep. It might be worth Dr. Whyte's while to experiment with oxygen or some oxygen carrier, with a view to mitigating the evil which his researches have indicated.

### Luxation of the Ulnar Nerve.

A Grenadier was admitted to hospital complaining of violent and increasing pain in the epitrochlear region, which radiated along the whole length of the internal border of the forearm into the ring and little fingers. Pain was increased by movements of flexion of the elbow-joint. In examining the limb, Dr. Gruner, according to the *Mediz. Klin.*, came across a nerve bundle, indurated and extremely painful in the bicipital groove. This he found to be the ulnar nerve which had slipped out of place. No history of recent injury could be obtained to account for the condition, but the patient was able to call to mind that several months previously he had struck his elbow against a wall. The injury had yielded to treatment by rest for a few weeks, and he was afterwards able to carry out his duties, and it was only little by little that the pains of which he now complained had grown unbearable. It was an easy matter to find the nerve and to put it back into place. The case is interesting as showing the slow development of symptoms following accidental displacement of a nerve-trunk.

### Non-pathogenic Oyster-beds.

In an article in the *Revue Médicale de Louvain*, under the heading "Official Investigations," Professor Ide maintains that official tests should be continued in the matter of the fitness of uncooked oysters for human food, even when no scare exists to stimulate activity in the matter. It has been definitely established that in a great number of cases oysters act as carriers of disease germs, and no difference in that respect is to be found between those coming from Belgium and those from France and Holland. The oyster receives twice daily a certain quantity of river or harbour water. It is sent away alive after having received a douche of dirty water within twelve hours. It is often sold and even eaten within forty-eight hours of being gathered. It thus has no chance of getting rid of its typhoid, para-typhoid B, or cholera germs before being consumed. In Italy several cases of typhoid fever contracted in this way were reported last winter, which had been preceded by an attack of para-typhoid fever from which the patients had seemingly recovered. It was shown that their serum agglutinated the organisms of both diseases. Under present conditions as regards the maintenance of the oyster-beds, the only oyster-eater who is safe from an attack of fever is he who happens to have been immunised by a previous attack. The author points out that it is ridiculous to allow the public to eat oysters produced under such conditions in an age when no one would dream of drinking unboiled water collected from a badly-constructed well. A fortune, he says, awaits the man who succeeds in constructing oyster-beds guaranteed free from infection, and in order to be able to do this the dirty soft water which the oyster needs for fattening purposes must be freed from typhoid and cholera germs. It may, by the help of ultra-violet rays or of ozone, be found possible to effect this at small cost, or even maybe, by the employment of better water or of that which has been exposed to sunlight for several days. Once done, it would be a simple matter to guard against carelessness on the part of middlemen.

### The Disadvantages of a Sugar Diet.

In commenting on the increasing consumption of sugar, *American Medicine* remarks that the attention of physiologists in many parts of the world has been directed to the fact, and that their opinion is on the whole favourable, because it is economical to relieve the digestive organs of part of their labours in digesting starch. The journal considers that there is no reason for fearing an atrophy of the starch-reducing apparatus, for such a result would need many generations for its accomplishment, and in any case the production of sugar is rapidly becoming sufficient to replace starches in food. There is, however, a recognised danger in the indiscriminate use of sugar. When starch is reduced by digestive processes, the sugar resulting is produced in weak solution, whereas when sugar is eaten as such it is taken into the system in strong solutions which are more or less harmful. Very strong solutions of sugar are used in nature as well as in the kitchen to preserve foodstuffs, for they are injurious to living tissue, being antiseptic. It is now said that sugar-eaters are more subject to inflammatory disorders of the liver and intestinal tract than are the rest of humanity. It would therefore seem necessary to warn the public that, in spite of a sugar diet being highly praised by some authorities, there is a danger in undue indulgence in sugar.

### The Psychopathic Constitution in Recruits.

A paper by Staff-Physician Stier in the *Berlin. Klin. Woch.* draws attention to a new departure in the processes whereby undesirable recruits and soldiers are weeded out of the German Army. It has been recognised that the possession of a psychopathic taint renders a man less amenable than the average to military discipline. Psychological methods of examination have therefore been adopted in addition to those already in use. Neurasthenia is the commonest of the psychopathic conditions and is recognised by the morbid irritability of the patient, his proneness to mental and physical exhaustion, and the possession of hyperæsthesia, headache and sleeplessness. Hysteria is characterised by inattention and lack of ideation. Traces of physical degeneration are more commonly met with than in neurasthenia. The hysterical class should be rejected at sight, but the neurasthenic can in some cases be accepted. The epileptic tendency is shown by ungovernable temper and intolerance of alcohol. The hereditary psychopathic taint produces changes in the emotions, conduct of life and personality, and may coexist with good mental powers as well as with physical stigmata. Physical is naturally closely related to moral degeneration, and attention should be paid as much to moral as to physical stigmata. The author does not consider misshapen ears to have much practical significance as stigmata of degeneration, but on the other hand malformations of the head, dental anomalies, peculiar distribution of the hair, and malformations of the genitalia are important. A disproportion between the candidate's age and his actual development is also good evidence of degeneration. Speech defects, squints, quivering of the eyelids, tics and habits such as biting the nails are taken as functional stigmata. This new departure on the part of the German military authorities will necessitate the revision of the manuals dealing with the examination of recruits, which at present only deal with the more pronounced physical and moral abnormalities.

